# Household Debt, Maternal Well-Being, and Child Adjustment in Germany: Examining the Family Stress Model by Family Structure

**DOI:** 10.1007/s10834-021-09777-1

**Published:** 2021-07-06

**Authors:** Valerie Heintz-Martin, Claudia Recksiedler, Alexandra N. Langmeyer

**Affiliations:** German Youth Institute, Nockherstr. 2, 81541 Munich, Germany

**Keywords:** Financial indebtedness, Family relations, Mental health, Coparenting, Structural Equation modeling

## Abstract

The amount of household debt tripled globally over the last decades and a sizable share of individuals and families are overindebted due to mortgages, credit cards, or consumer debt. Yet research on the distribution of debt across families, and potential ripple effects of the psychological burden related to debt on well-being and family relations, remains sparse. Our study aims to fill these gaps by examining the socio-demographic profiles of families that have accumulated household debt and the unique role that the psychological burden related to debt plays on associations between mothers’ well-being, parental dynamics, and child adjustment based on the Family Stress Model (FSM). We used representative survey data collected in 2019 from Germany (*N* = 3271), which is one of the richest economies worldwide, yet about 10% of adults reported to be overindebted. Logistic regression results showed that single mothers were less likely to have debt compared to mothers in two-parent families. However, both single mothers and mothers in stepfamilies with high levels of perceived economic strain were particularly likely to report having debt. Structural equation modeling yielded that the links between the psychological burden of debt, maternal well-being, parental dynamics, and child adjustment were largely in line with the FSM, except for single mothers. We conclude that persisting financial disparities by family structure may be partially fostered by unique characteristics of the German welfare state, such as promoting more a traditional two-parent norm, and discuss our findings in light of practical implications.

From the 1990s until the global *Great Recession* of 2007, the amount of household debt rose globally and stagnated subsequently. Debt is hereby defined as all financial liabilities of households that require payments to creditors at a fixed date in the future (Organisation for Economic Co-operation and Development [OECD], 2020). Households in social democratic and liberal welfare states tend to accumulate more debt compared to households in conservative welfare states (Coletta et al., 2019). But even in Germany, which operates under a conservative welfare state and is among one the richest countries in the world (World Population Review, 2020), about 10% of individuals aged 18 years and over were *overindebted* in 2019 (STATISTA, 2020). This indicates that the accumulated amount of debt reached a point where overindebted individuals are no longer able to fulfill their financial obligations or have to skip payments to creditors (Federal Ministry of Labour & Social Affairs, 2017).

In addition to the sizable share of overindebted individuals, an increasing number of families with and without young children report having debt due to mortgages, credit cards, or consumer debt (Berger et al., 2016). Prior research documented the broader link between families’ financial strain, the quality of relationships within families, as well as child and parental well-being without focusing on debt per se (Conger & Conger, 2002; Heintz-Martin & Langmeyer, 2019; Lopoo & DeLeire, 2014; Nomaguchi & Milkie, 2020; Stack & Meredith, 2018). Yet research on the distribution of household debt across families, as well as how the accumulation of household debt affects individual family members and families as a whole, remains sparse (Dwyer et al., 2011; Mascher & Damberger, 2012; Sweet et al., 2013). This is particularly striking in light of constantly high rates of child poverty affecting 20% of children in Germany in 2017 (Federal Ministry for Family Affairs, Senior Citizens, Women and Youth, 2017) and their strong association with adverse proximal and long-term effects on the health and well-being of families and children (Cheval et al., 2019; Duncan et al., 2012; Hayward & Gorman, 2004; Masarik & Conger, 2017; Shonkoff & Garner, 2012; Shonkoff et al., 2009).

To fill these research gaps, the aims of our study are twofold. First, we examine the distribution of debt across families by family structure because rates of economic strain are particularly high among post-separation families (Bernardi & Mortelmans, 2018; Chzhen & Bradshaw, 2012; Raley & Sweeney, 2020). Second, we focus on the adverse ripple effects household debt may have on families by examining the unique pathways between the perceived psychological burden related to having household debt and parents’ well-being, their parenting practices, and child adjustment. Again, we further probe whether adverse links between the perceived psychological burden related to having household debt and strained family processes may be more pronounced among the more vulnerable, financially-strained group of post-separation families.

## Financial and Personal Strain Due to Debt

Since the 1990s, rates of consumer debt related to credit card debts or personal borrowing grew faster than consumers’ gross monthly income in the United States (US) and many other OECD countries (Balestra & Tonkin, 2018). Consequently, the average amount of household debt has tripled over the last decades and a larger share of individuals is overindebted (Sweet et al., 2013). The spread and amount of household debt rose for several reasons. First, cultural and technological shifts, such as the wide availability and convenience of online shopping, have led to increased rates of consumption in many domains of life. Second, the average costs of living have increased substantially in some areas, such as paying for housing in urban centers (Federal Statistical Office, 2020; Weber, 2018). In addition and especially in North America, deregulation of the financial sector loosened credit constraints (Hurst, 2011), which heightened individuals’ risk to accumulate unsecured and revolving credit card debt and purchases through installment plans (Berger et al., 2016; Xiao & Yao, 2011).

There are different kinds of debt. Long-term debt, such as buying a house, is often an investment in the future for one’s retirement or as inheritance for one’s children (Aratani & Chau, 2010). Short-term debt is often made in order to pay for daily goods, smaller consumer goods, sudden, more pricey purchase, which tend to be more common in lower-income and financially-strained households (Pfeiffer et al., 2016). The degree to which families are affected by financial strain has shown to vary considerably by family structure (e.g., Bernardi & Mortelmans, 2018; Brady & Burroway, 2012; Heintz-Martin & Langmeyer, 2019) through at least two mechanisms (Umberson & Thomeer, 2020). First, and due to social selection into marriage, healthier and wealthier individuals are more likely to marry and to remain married. Second, and in line with social causation, marriage can contribute to differences in wealth over time because of the joint accumulation of financial assets and the forgone costs of union dissolution compared to single parents and stepfamilies. Thus, post-separation parents could be also more likely to take on short-term debt and the gap between those who are able to take on debt for investment purposes and those who supplement insufficient income with debt for daily consumption has widened over the past decades (Cooper & Pugh, 2020), particularly during the Great Recession (Dunn & Mirzaie, 2016; Jenkins et al., 2013).

Even though incurring debt to accumulate assets can be beneficial, it can also lead to financial pressure simply because the money must be paid back eventually (Berger et al., 2016). Findings on the link between debt and psychological well-being are somewhat mixed. Some studies found a negative, but indirect link between debt and the well-being of individuals (Aratani & Chau, 2010; Berger et al., 2016; Dunn & Mirzaie, 2016; Sweet et al., 2013). Dew (2007, 2011) further reported differences in marital satisfaction and levels of spousal conflict depending on the amount of debt. Some argued that financial stress due to debt is related to lower psychological functioning (Brown et al., 2005) and a higher risk to experience mental disorders (Jenkins et al., 2008), such as anxiety (Drentea, 2000; Drentea & Reynolds, 2012) or depression (Bridges & Disney, 2010; Drentea & Reynolds, 2012; Gathergood, 2012). However, Dew (2007) suggested that debt lowered the risk of depression among married couples and others found even positive links between self-esteem and debts (Dwyer et al., 2011), which is likely due to social disparities related to differences in individuals’ reasons for taking on debt.

## Financial Strain in Families

A large body of research has focused on the welfare of low-income families compared to their more affluent counterparts (Cooper & Pugh, 2020). The well-established Family Stress Model (FSM) (Conger & Conger 2002; Conger et al., 2010) is a particularly useful theoretical framework to explain the influence of economic hardship on the well-being of parents and children. The key assumption of the FSM is that financial strain represents a psychological burden that influences parents’ emotional well-being, which then has an adverse effect on and the quality of family interactions (Waylen & Stewart-Brown, 2010). Interactions between family members, such as parents’ ability to work together as a team with regard to parenting practices (hereafter, ‘coparenting’), has also shown to vary by socioeconomic factors (e.g., income; McDaniel & Teti, 2012). This can, in turn, lead to increased levels of child behavioral problems (Conger & Conger, 2002; Conger et al., 2010). The strength of the FSM lies in its ability to account for micro-level stress processes due to economic hardship among individual family members and on families as a whole, as well as its potential ripple effects for children.

Even though the FSM has not specifically been applied to the burden related to having household debt to our knowledge, several studies examined the links between maternal depression, conflictual coparenting, and harsh parenting practices more broadly. Some findings indicated that a more positive coparenting relationship contributes to the well-being of parents and that more negative coparenting relationship is a predictor of depressive symptoms for mothers and fathers (Solmeyer & Feinberg, 2011). Others found evidence that maternal depression was a predictor of parental coparenting (McDaniel & Teti, 2012). Tissot and colleagues (2017) argued that the relationship between parental depression and coparenting is reciprocal. Their recent longitudinal study suggested that depressive symptoms were more likely to affect the quality of the parental coparenting relationship than the other way around (Tissot et al., 2017). Similarly, Williams (2018) found that parental depression was associated with decreased levels of cooperative coparenting. A recent study by Choi and Becher (2019) further showed that maternal depression was positively associated with harsh parenting practices, which, in turn, increased the likelihood of child behavioral problems.

The FSM has also been replicated among a diverse set of ethnic backgrounds and geographic locations (for an overview of empirical studies using the FSM, see Masarik and Conger, 2017), yet it has rarely been examined whether differences by family structure exist (Schramm & Adler-Baeder, 2012). Even though two-parent families are less likely to experience financial strain compared to post-separation families (Chzhen & Bradshaw, 2012; Dziak et al., 2010), it could also be the case, that when affected by it, couples in two-parent families might cope differently with financial strain. Due to the complex structure and larger size of stepfamilies, more family members are involved in decisions concerning financial issues and they may need more disposable income to cover their basic needs and additional expenses (e.g., child support payments; Coleman et al., 2001; Heintz-Martin & Langmeyer, 2019; Malone et al., 2010; Stewart, 2001). For single parents, who already suffer from large penalties in life satisfaction, mental and physical health compared to two-parent families (Dziak et al., 2010; Hurst, 2011), particularly in less generous welfare states (Burstrom et al., 2010; Pollmann-Schult, 2018), dealing with financial strain may have even more adverse effects on their well-being (Stack & Meredith, 2018) and the quality of the parent–child relationship (Waylen & Stewart-Brown, 2010).

## The Present Study

Our study focuses on Germany because, despite being among one of the richest countries in the world, it has a rather high poverty rate with roughly 17% of its population being poor in 2016 (Aust et al., 2018). Among those affected by poverty, single parents (about 40%) and families with three or more children in their household (about 30%) were overrepresented (Aust et al., 2018). Disparities in financial strain among post-separation families may also be fostered by the fact that Germany operates under a male-breadwinner model, which actively discourages both parents to work through taxation leaving little fiscal benefit for dual-earners and promoting a more traditional two-parent norm (Grunow et al., 2018; Thévenon, 2011). About 10% of Germans aged 18 years and over were further overindebted (STATISTA, 2020) and at least one child was living in about 35% of the overindebted households in 2019 (Federal Statistical Office, 2020). Compared to the US, credit card debt is not the major reason for debt accumulation in Germany because access to credit cards is more strongly regulated. However, rates of consumer debt rose steadily—especially for low-income households—due to the availability of installment purchases (Pfeiffer et al., 2016) and increased housing costs (Federal Statistical Office, 2020). Other risk factors associated with the risk of being overindebted in Germany were union dissolution (12.5%) or insufficient financial literacy (14.3%; Federal Statistical Office, 2020).

Against this backdrop, our study on the distribution across and impact of household debt on families has two aims. First, we aim to examine the distribution of debt across families by family structure in Germany, which is a particularly timely issue because the amount of household debt is rising globally. We expect single parents and stepfamilies compared to two-parent families to be more likely to report having household debt (*Hypothesis 1a*) because of their more precarious economic situation. We also expect both individuals with low and high levels of perceived economic strain to be more likely to report having household debt (*Hypothesis 1b*), even though the reasons for taking on debt are likely to differ between these groups (i.e., future investments vs. daily consumption). Lastly, we expected both single parents and stepfamilies with high levels of perceived economic strain to be more likely to report having debt compared to two-parent families with high levels of economic strain (*Hypothesis 1c*) because of the costs associated with union dissolution and the high load of stressors that post-separation families are exposed to.

**Fig. 1:**
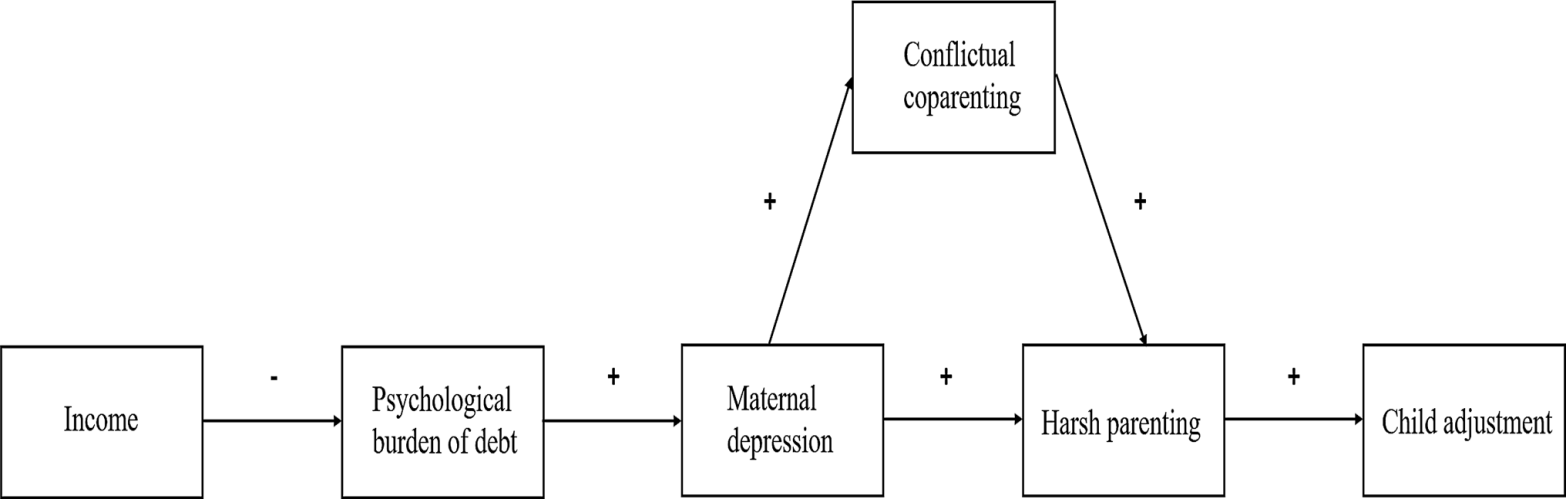
Conceptual model of the links between the perceived burden of debt, maternal well-being, parenting practices, and child adjustment. Adapted from the Family Stress Model (Conger & Conger, 2002; Conger et al., 2010)

Second, we aim to examine the adverse ripple effects of household debt on families by tracing pathways from families’ perceived psychological burden due to debt on parental well-being, the quality of relationships within families, and child adjustment. Drawing on the FSM (see conceptual model in Fig. 1; *Hypothesis 2a*), we expect a lower income to be related to a higher psychological burden due to debt, which can, in turn, be associated with higher levels of maternal depression. We further anticipate to find a positive link between maternal depression and conflictual coparenting between both parents, as well as between maternal depression and mothers’ harsh parenting practices. Lastly, we also expect conflictual coparenting to be also associated with harsher parenting practices, which can, in turn, increase the risk of adverse child adjustment. Because the ripple effects of household debt on families may vary systematically by family structure, we expect the adverse link between the perceived burden of debt, strained family processes, and detrimental child adjustment to be more pronounced among single parents and stepfamilies compared to two-parent families (*Hypothesis 2b*).

## Method

### Data

We used data from the third and most recent installment of the large-scale, representative German survey “Growing up in Germany,” which was collected via standardized computer-assisted interviews in 2019. A sample of 0 to 32 year-olds was drawn in two steps. First, municipalities across Germany were sampled with inclusion probabilities proportional to the number of inhabitants. Second, a fixed-sized sample of individuals within the target age range was randomly drawn from the municipalities’ population registers. These target persons, or the primary caretaker for minors, were then contacted by professional interviewers to schedule an appointment for the interview. Once target persons or their primary caretakers agreed to participate in the study, data were not only collected from the target person, but modularized interviews were also conducted with other members of the household (e.g., parents or siblings), pending their willingness to take part in the study as well. The survey covered a wide range of topics, such as the socio-economic circumstances of families and individual family members, family processes, and indicators of well-being. Participants received a small compensation for taking part in the study and the response rate was 21% of households with target persons initially drawn from the population registers. The full sample included 14,277 interviews with persons aged 0–32 years and 6621 parent interviews for minors both nested in 6355 households.

For the purpose of this study, we restricted our subsample to mothers with children aged 4 to 17 years in their households because responses on key indicators in our analyses were only provided by mothers (e.g., household debt) and for children of these ages (e.g., child adjustment). Another reason to select mothers only was our focus on disparities by family structure, which vary substantially by gender (Bernardi & Mortelmans, 2018). For example, the vast majority of single-parent households were female-headed in our data (about 87%). Our final analytical sample consisted of 3271 mothers in two-parent families (about 76%), single-parent families (about 14%), and stepfamilies (about 10%).

### Measures

#### Families’ Socioeconomic Situation

Three indicators of families’ socioeconomic situation were available, which we used at different steps of the analyses (see Analytical Strategy below). First, mothers were asked whether their *household had “accumulated debt or running loans”* (hereafter, ‘household debt;’ 0 = no; 1 = yes). If respondents indicated to have household debt, mothers were further asked to rate to which degree they perceive the accumulated *household debt as a psychological burden* (1 = not a burden; 2 = somewhat of a burden; 3 = a considerable burden).

Second, we have information on families’ equalized monthly net *household income* using modified OECD equivalence weights (in Euros). Note that we entered the logged equalized household income into the models because of its skewed distribution.

Finally, families’ level of *perceived economic deprivation*, was assessed with three indicators. Respondents were asked to indicate whether the following statements applied to their financial situation (1 = yes; 2 = no because of financial reasons; 3 = no because of other reasons): “We can put away money each month,” “We can replace furniture,” and “We can pay for unexpected expenses.” We collapsed negative replies into one category and formed a count of these answers as indicator of perceived economic deprivation in our models (1 = none; 2 = low, i.e., count of one; 3 = high, i.e., count of two or three).

#### Psychological Distress

Maternal *depressive symptoms* were assessed using the WHO-5 scale (Topp et al., 2015). Mothers were instructed to rate how often they felt the following emotions during the last two weeks on a scale from 1 (at no time) to 6 (all the time): cheerful and in good spirits; calm and relaxed; active and vigorous; woke up fresh and rested; daily life filled with things that interested me. Items were recoded so that higher values indicated higher levels of depressive symptoms and the internal consistency of the scale was good (Cronbach’s α = 0.82).

#### Quality of Coparenting

Mothers were asked to rate the quality of the *coparenting relationship with the other parent* of a given child by rating six indicators, such as “We are a good team as parents “ or “Discussions about parenting practices often end with us fighting,” from the Parent Problem Checklist (Dadds & Powell, 1991) on a scale from 1 (completely disagree) to 6 (completely agree). Items were recoded so that higher values indicated a poorer quality of coparenting and the internal consistency of the scale was good (Cronbach’s α = 0.80).

#### Parenting Practices

Mothers were asked to rate the following three statements concerning their own *harsh parenting practices* on a scale from 1 (never) to 6 (always): “I punish my child harsher than it deserves,” “I punish my child harshly, also for minor mishaps,” and “I get angry easily when my child does not do what I say.” Items were recoded so that higher values indicated harsher parenting practices and the internal consistency of the scale was satisfactory (Cronbach’s α = 0.70).

#### Child Adjustment

Children’s *psychosocial adjustment* was measured by the Strength and Difficulties Questionnaire (SDQ; Goodman, 2001), which is a validated method to assess child and youth problem behavior for minors between the Ages 4 to 17 years. The SDQ consists of five subscales (i.e., emotional problems, conduct problems, hyperactivity, peer problems, and prosocial behavior; 0 = not true; 1 = somewhat true; 2 = certainly true) with five items each and a total difficulty score (TDS) is formed by summing up scores from all subdimensions except prosocial behavior scale (see www.sdqinfo.org for more information on the scoring of the SDQ). In our study, the SDQ was administered to mothers, who rated these indicators for one child in the household (if there was more than one child in the household). In most cases, this was the oldest child in the household.

#### Sociodemographic Information

Information on mothers’ and children’s age (in full years), the number of children in the household (from 1 = 1 child, to 3 = 3 or more children), mothers’ educational attainment based on the Comparative Analysis of Social Mobility in Industrial Nations index (Brauns et al., 2003; 1 = primary, to 3 = tertiary) and employment status (1 = not employed, i.e., unemployed, in post-secondary training, or on parental leave; 2 = marginally or part-time employed; 3 = full-time employed), whether mothers were migrants (i.e., one or both of mothers’ parents, or the mother herself, were born in another country) or native-born (0 = no; 1 = yes), and whether families received or provide financial support from or to kin (2 items; 0 = no; 1 = yes) were available and served as control variables at certain steps of the analytical strategy (see below).

### Analytical Strategy

All analyses were conducted in Stata (v15.1). To address our first research aim on the distribution of debt across families, we used logistic regression models predicting whether families had accumulated household debt. We entered the predictors and control variables into the model in two steps. In Model 1, we first entered the main effects of our predictors (i.e., family structure and families’ levels of perceived economic deprivation) and controls (i.e., mothers’ age, educational attainment, employment status, and migrant status; the age of the youngest child and the number of children in the household; families’ monthly income and financial support from/to kin) to examine whether the likelihood of having household debt varied by family structure and families’ level of economic deprivation. Model 2 then also included interaction terms between family structure and levels of perceived economic deprivation to examine whether particularly post-separation families with a higher load of stressors (i.e., perceived economic deprivation) were more likely to report having household debt. To ease the interpretation of significant interaction terms, we estimated and plotted predictive margins.

In order to address our second research aim on the ripple effects of household debt on families and by family structure, we used Structural Equation Modeling (SEM; Acock, 2013; Kline, 2016). This method is particularly suited to investigate path dependencies between a range of constructs simultaneously, as outlined in our conceptual model, rather than running several separate regression models consecutively. Another key advantage of SEM compared to other ordinary regression model is the availability of goodness of fit measures in SEM (see below), which allow to evaluate how well an estimated model fits the observed data. Furthermore, the use of latent variables for multi-item indicators in SEM models, compared to manifest predictors only in ordinary regression models, reduces measurement error in the estimates (Card & Little, 2007).

Our SEM models were estimated based on the subset of respondents who reported having household debt only (*N* = 2134; 66.3% of the sample) and in two steps. We first fitted a model based on this subsample as a whole and, second, fitted a multi-group analysis (MGA) stratified by family structure to examine whether these processes vary systematically between two-parent families, single-parent families, and stepfamilies. Because our conceptual model also included mediated paths between maternal depressive symptoms, conflictual coparenting, and mothers’ harsh parenting practice, we estimated and tested all indirect effects to test for mediation both in the overall model and MGA (Hayes, 2009).

As goodness of fit measures for both models served the chi-square value of the model, the comparative fit index (CFI; should be at least 0.90), the root mean squared error of approximation (RMSEA; should be at least 0.08 or less), and the standardized root mean squared residual (SRMR; should be less than 0.08; Acock, 2013). Standardized coefficients are reported for all outcomes and we used a full information maximum likelihood approach in our SEM models to account for missing data. This approach does not impute missing values, but uses all available information in its maximum likelihood estimation (Acock, 2012; Enders, 2010).

## Results

### Descriptive Results

Table 1 displays summary statistics of key study variables by family structure. Significant group differences by family structures on these indicators were probed with chi-squared tests for categorical indicators and analysis of variance for continuous indicators. It can be seen that, among stepfamilies compared to two-parent families and single parents, the age of the youngest child in the household tended to be slightly younger and the share of families with three or more children considerably higher (about 48% vs. about 30% and 24%, respectively). In contrast, the largest share of families with only one child was higher for single parents compared to two-parent and stepfamilies (about 38% vs. about 15% and 13%, respectively). With regard to mothers’ educational attainment, a higher share of single mothers and mothers in stepfamilies had only primary levels of schooling compared to mothers in two-parent families (about 24% and 26% vs. 13%, respectively), and more than half of mothers in two-parent families held tertiary levels of schooling.

**Table 1:** Descriptive sample statistics by family structure

Indicators	Total	Two-parent families	Single parents	Stepfamilies
*N* (%)	3271	2479(75.8)	469 (14.3)	323 (9.9)
Age of mother, *M* (*SD*)	*(F* = *11.60, df* = *2; p* < *0.001)*			
	40.31 (6.96)	40.50 (6.73)^a^	40.56 (8.04)^b^	38.55 (6.76)^a,b^
Age youngest child, *M* (*SD*)	*(F* = *31.54, df* = *2; p* < *0.001)*			
	7.31 (4.80)	7.18 (4.75)^a,b^	8.76 (4.82)^a,c^	6.20 (4.73)^b,c^
Number of children, *n* (%)	*(Chi*^*2*^ = *180.85, df* = *4; p* < *0.001)*			
1 child	593 (18.1)	375 (15.1)	176 (37.5)	42 (13.0)
2 children	1662 (50.8)	1355 (54.7)	180 (38.4)	127 (39.3)
3 or more children	1016 (31.1)	749 (30.2)	113 (24.1)	154 (47.7)
Education, *n* (%)	*(Chi*^*2*^ = *124.81, df* = *4; p* < *0.001)*			
Primary	514 (15.9)	320 (13.0)	112 (24.1)	82 (25.9)
Secondary	1077 (33.2)	762 (31.0)	174 (37.4)	141 (44.5)
Tertiary	1647 (50.9)	1374 (56.0)	179 (38.5)	94 (29.6)
Employment status, *n* (%)	*(Chi*^*2*^ = *61.94, df* = *4; p* < *0.001)*			
Not employed^1^	1003 (30.7)	754 (30.4)	135 (28.9)	114 (35.4)
Marginal/Part-time	1610 (49.3)	1295 (52.3)	191 (40.8)	124 (38.5)
Full-time	654 (20.0)	428 (17.3)	142 (30.3)	84 (26.1)
Migrant, *n* (%)	*(Chi*^*2*^ = *4.50, df* = *4; p* = *0.10)*			
	881 (27.1)	690 (27.9)	119 (25.6)	72 (22.6)
Monthly HH income, *M* (*SD*)	*(F* = *107.07, df* = *2; p* < *0.001)*			
	1851.10 (1569.70)	2006.67 (1664.94)^a,b^	1253.67 (1082.40)^a,c^	1554.95 (1126.25)^b,c^
Economic deprivation, *n* (%)	*(Chi*^*2*^ = *236.82, df* = *4; p* < *0.001)*			
None	2287 (70.4)	1887 (76.5)	202 (43.4)	198 (61.9)
Low	518 (15.9)	336 (13.6)	118 (25.4)	64 (20.0)
High	446 (13.7)	243 (9.9)	145 (31.2)	58 (18.1)
Receiving financial	*(Chi*^*2*^ = *44.66, df* = *4; p* < *0.001)*			
Assistance from kin, *n* (%)	489 (15.1)	313 (12.8)	109 (23.6)	67 (20.9)
Providing financial	*(Chi*^*2*^ = *5.01, df* = *4; p* = *0.08)*			
Assistance for kin, *n* (%)	368 (11.4)	285 (11.6)	40 (8.6)	43 (13.4)
HH debt, *n* (%)	*(Chi*^*2*^ = *25.50, df* = *4; p* < *0.001)*			
	2134 (66.3)	1645 (67.5)	261 (56.4)	228 (71.2)
Burden due to debt, *n* (%)	*(Chi*^*2*^ = *61.01, df* = *4; p* < *0.001)*			
None	537 (25.3)	448 (27.3)	37 (14.3)	52 (23.1)
Low	1079 (50.7)	856 (52.1)	112 (43.2)	111 (49.3)
High	510 (24.0)	338 (20.6)	110 (42.5)	62 (27.6)
Depression, *M* (*SD*)	*(F* = *28.68, df* = *2; p* < *0.001)*			
	2.96 (0.87)	2.90 (0.84)^a,b^	3.19 (0.95) ^a^	3.11 (0.93)^b^
Coparenting, *M* (*SD*)	*(F* = *209.65, df* = *2; p* < *0.001)*			
	2.00 (0.97)	1.86 (0.84)^a,b^	2.93 (1.30)^a,c^	2.12 (0.97)^b,c^
Harsh parenting, *M* (*SD*)	*(F* = *6.25, df* = *2; p* < *0.01)*			
	1.78 (0.64)	1.80 (0.64)^a^	1.68 (0.58)^a,b^	1.79 (0.69)^b^
Child adjustment, *M* (*SD*)	*(F* = *41.59, df* = *2; p* < *0.001)*			
	8.63 (5.14)	8.19 (4.76)^a,b^	9.75 (5.79)^a^	10.45 (6.20)^b^

*HH* Household

^1^includes mothers in post-secondary training and on parental leave. Range: age parents (18–68 years); age youngest child (0–17 years); number of children (1–6); monthly household income (75–16,875 Euros); depression (1–6); coparenting quality (1–6); harsh parenting (1–6); child adjustment (1–34). For continuous indicators, identical superscripted letters indicate significant differences between the respective groups

The majority of all mothers worked part-time or were only marginally employed, yet a considerable share of single mothers was full-time employed particularly compared to mothers in two-parent families (about 30% vs. about 17%, respectively). The share of mothers with migrant status, however, did not vary significantly by family structure. Families’ monthly net income was lowest among single parents and the share of families receiving assistance from kin was also the highest for single parents compared to two-parent and stepfamilies. The share of families with high levels of perceived economic deprivation was the highest among single parents compared to two-parent and stepfamilies (about 31% vs. about 10% and 18%, respectively). Yet the share of families reporting to have household debt was higher among both stepfamilies and two-parent families compared to single parents. Among single parents with accumulated household debt, however, the share of parents reporting a high psychological burden due to debt was highest for single parents as well compared to two-parent and stepfamilies (about 43% vs. about 21% and 28%, respectively).

### Results from the Logistic Regression Models

Table 2 shows the results from the logistic regression models predicting the likelihood of having debt. In Model 1, only single parents, but not mothers in stepfamilies, were significantly less likely to report having debt compared to two-parent families. Mothers with secondary levels of schooling were more likely to report having debt compared to those with primary levels of schooling, as well as those with part- or full-time employment compared to mothers outside of the labor market (including mothers still in training or on parental leave). Mothers supporting other relatives financially, but not those who received financial support from kin, were more likely to report having debt. Income was also positively and mothers’ migrant status negatively associated with the risk of having household debt. Yet families reporting both lower and higher levels of perceived economic deprivation were also more likely to have debt compared to those who were not economically deprived.

**Table 2:** Results of logistic regression models predicting the likelihood of having household debt

Predictors	Model 1	Model 2
Intercept	0.06 (0.04)^***^	0.07 (0.04)^***^
Single parents ^a^	0.66 (0.08)^**^	0.52 (0.09)^***^
Stepfamilies ^a^	1.12 (0.16)	0.95 (0.17)
Age of mother	0.99 (0.01)	0.99 (0.01)
Age youngest child	0.98 (0.01)	0.99 (0.01)
Two children in HH ^b^	1.10 (0.12)	1.09 (0.12)
Three or more children in HH ^b^	1.16 (0.15)	1.19 (0.16)
Secondary education ^c^	1.67 (0.21)^***^	1.70 (0.21)^***^
Tertiary education ^c^	1.13 (0.14)	1.15 (0.15)
Part-time employed ^d^	1.45 (0.15)^***^	1.45 (0.15)^***^
Full-time employed ^d^	1.49 (0.19)^***^	1.52 (0.20)^**^
Migrant	0.65 (0.06)^***^	0.66 (0.06)^***^
Monthly HH income	1.64 (0.13)^***^	1.62 (0.13)^***^
Receiving support from kin	1.13 (0.18)	1.13 (0.13)
Providing support for kin	1.34 (0.18)*	1.34 (0.18)^*^
Low deprivation ^e^	1.63 (0.20)^***^	1.61 (0.24)^**^
High deprivation ^e^	1.66 (0.23)^***^	1.16 (0.19)
Single parents x Low deprivation		1.11 (0.32)
Single parents x High deprivation		2.52 (0.71)^**^
Stepfamilies x Low deprivation		1.23 (0.47)
Stepfamilies x High deprivation		2.33 (0.92)^*^

Cells show odds ratios and standard errors in brackets. *HH* Household. Reference category is: ^a^Single-parent families; ^b^One child in HH; ^c^Primary education; ^d^Not employed (i.e., unemployed, in education, or on parental leave); ^e^No perceived economic deprivation. ^*^p < 0.05. ^**^p < 0.01. ^***^p < 0.001

**Fig. 2:**
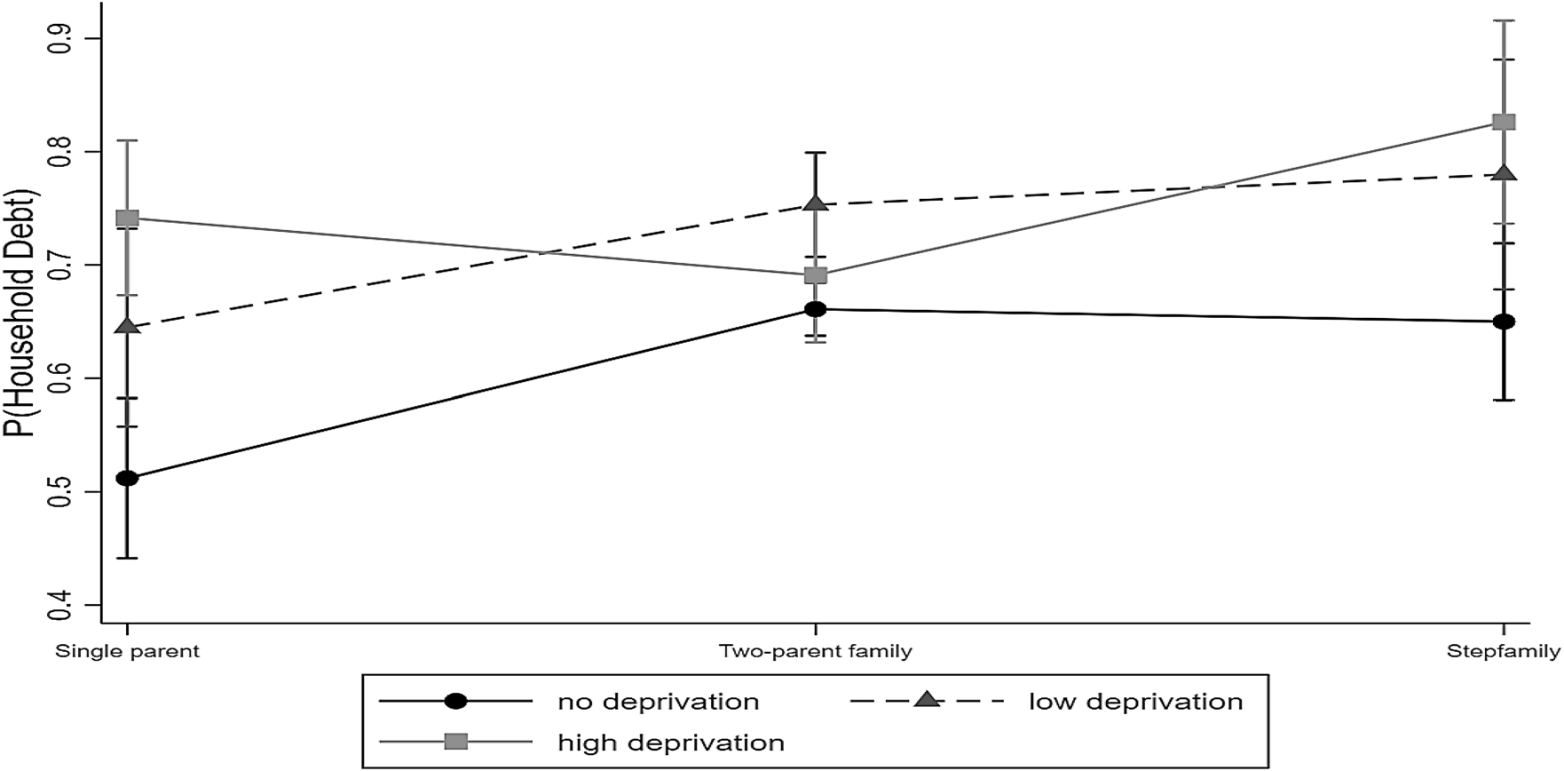
Predictive probabilities of the likelihood of having household debt by family structure and levels of perceived economic deprivation

In Model 2, significant interaction terms between both single parents and high levels of perceived economic deprivation, as well as between stepfamilies and high levels of perceived economic deprivation emerged. Figure 2 shows the predicted probabilities of the likelihood of having household debt by family structure and levels of perceived economic deprivation. It can be seen that respondents with no perceived economic deprivation had the lowest likelihood to report having household debt across family structure. Both single parents and stepfamilies with high levels of economic deprivation, however, were particularly prone to report having debt compared to two-parent families. Among two-parent families, those with low levels of economic deprivation had the highest risk of reporting to have household debt. There were no significant differences for the composition of the household (i.e., number of children and age of the youngest in the household) or mothers’ age in both models.

### Results from the Overall Structural Equation Model

**Fig. 3:**
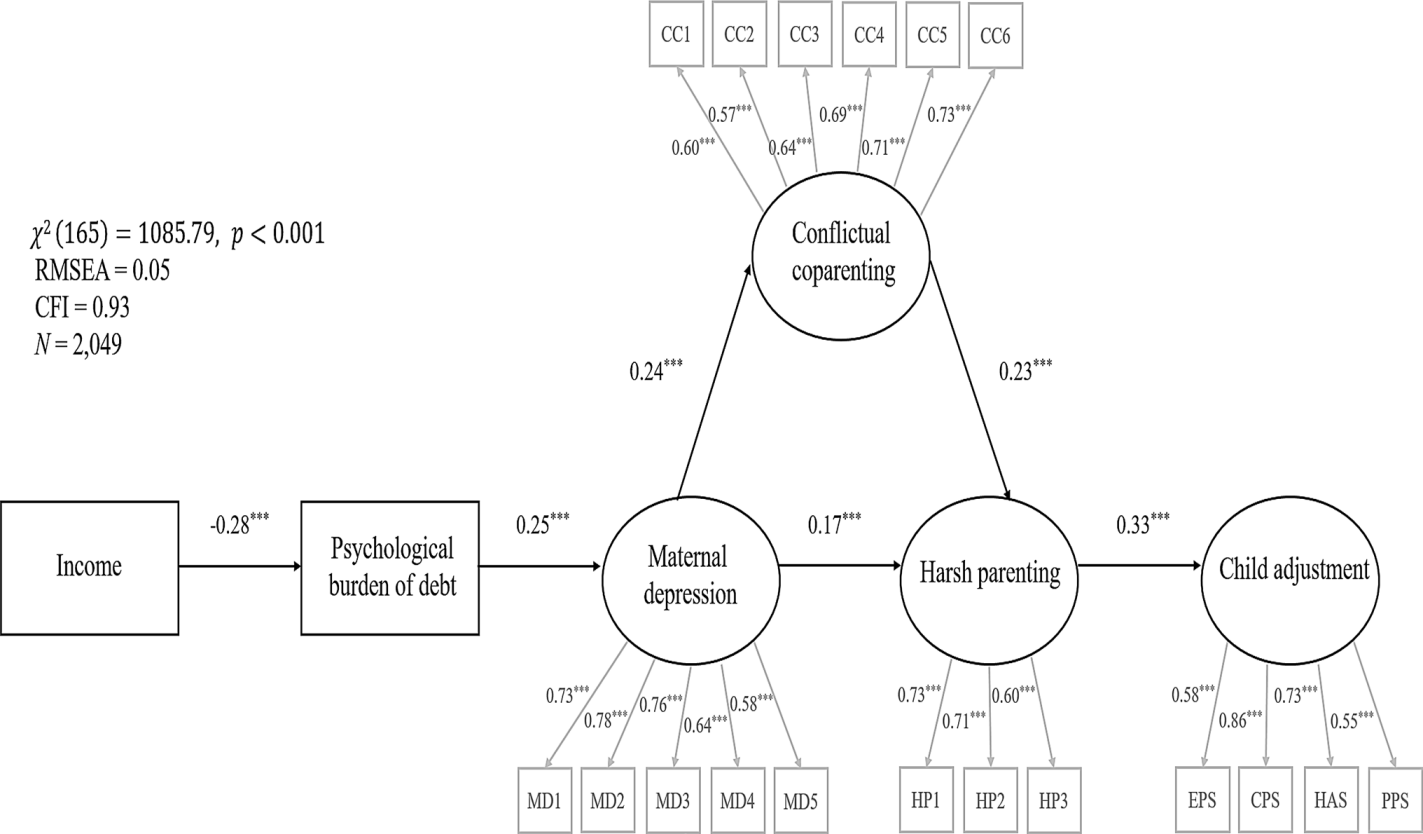
Structural equation model displaying standardized coefficients for the direct structural paths. Rectangles indicate manifest indicators; circles indicate latent constructs; error terms are omitted. *MD* Maternal depression, *HP* Harsh parenting, *CC* Conflictual coparenting, *EPS* Emotional problem subscale, *CPS* Conduct problem subscale, *HAS* Hyperactivity subscale, *PPS* Peer problem subscale. ^***^p < 0.001

Using the subsample of families with accumulated household debt (about 66% of the sample), we ran a SEM to quantify the relationships between the psychological burden related to having household debt, maternal well-being, (co-)parenting practices, and child adjustment. Note that in the measurement model (see Fig. 3), single-item indicators (i.e., income and the psychological burden related to having household debt) were entered as manifest constructs into the model and items of multi-item indicators (e.g., maternal depression or quality of the parental coparenting relationship) were entered individually to form latent constructs in the model. One exception was the latent construct of child psychosocial adjustment problems because, in line with prior studies (e.g., van den Eynde et al., 2020), this construct was formed by entering mean scores of the four subscales of the TDS (i.e., emotional problems, conduct problems, hyperactivity, peer problems).

Model fit indices and standardized direct structural path coefficients of the structural model are displayed in Fig. 3. The RMSEA smaller than 0.08 and the CFI larger than 0.90 indicated a good model fit. Results for the structural path coefficients were in line with our conceptual model. Lower-income was significantly associated with a higher psychological burden related to debt. A higher debt-related psychological burden was further positively related to mothers’ ratings of depressive symptoms. Higher levels of mothers’ depressive symptoms were linked to both higher levels of conflict concerning coparenting duties with the biological father and mothers’ harsher parenting practices. Higher levels of conflict concerning coparenting practices were associated with mothers’ harsher parenting practices as well, and subsequently, were linked to higher levels of adjustment problems of the child. Lastly, the coefficients for all indirect paths displayed in Table 3 were significant, which indicates that all mediation hypotheses specified in the conceptual model (e.g., between maternal depression, conflictual coparenting, and mother’s harsh parenting practices) were fulfilled.

**Table 3:** Indirect effects of the overall structural equation models and MGA

Outcome	Indirect effects			
	Overall	Two-parent families	Single parents	Step-families
Depression				
Income → Depression	−0.07^***^	−0.07^***^	−0.07^**^	−0.09^***^
Conflictual coparenting				
Income → Coparenting	−0.02^***^	−0.02^***^	−0.03^**^	−0.02^**^
Burden → Coparenting	0.06^***^	0.06^***^	0.14^***^	0.08^***^
Harsh parenting				
Income → Harsh	−0.02^***^	−0.02^***^	−0.01	−0.02^**^
Burden → Harsh	0.06^***^	0.07^***^	0.05^*^	0.08^***^
Depression → Harsh	0.06^***^	0.06^***^	0.09	0.09^**^
Child adjustment problems				
Income → Child adjustment	−0.01^***^	−0.01^***^	−0.01	−0.01^*^
Burden → Child adjustment	0.02^***^	0.02^***^	0.02	0.03^**^
Depression → Child adjustment	0.08^***^	0.09^***^	0.05	0.10^**^
Coparenting → Child adjustment	0.08^***^	0.09^***^	0.07	0.14^**^

Cells show standardized coefficients for the structural paths. ^*^p < 0.05. ^**^p < 0.01. ^***^p < 0.001

### Results from the MGA by Family Structure

We further stratified our models by family structure to test whether the strength of these associations varies systematically between two-parent families, single parents, or stepfamilies. A significant chi-squared test for the difference between the MGA with and without equality constraints across the groups indicated that the structural paths differed by family structure (χ^2^(12) = 62.56, *p* < 0.001; Acock, 2013; Pruett et al., 2003).

**Fig. 4:**
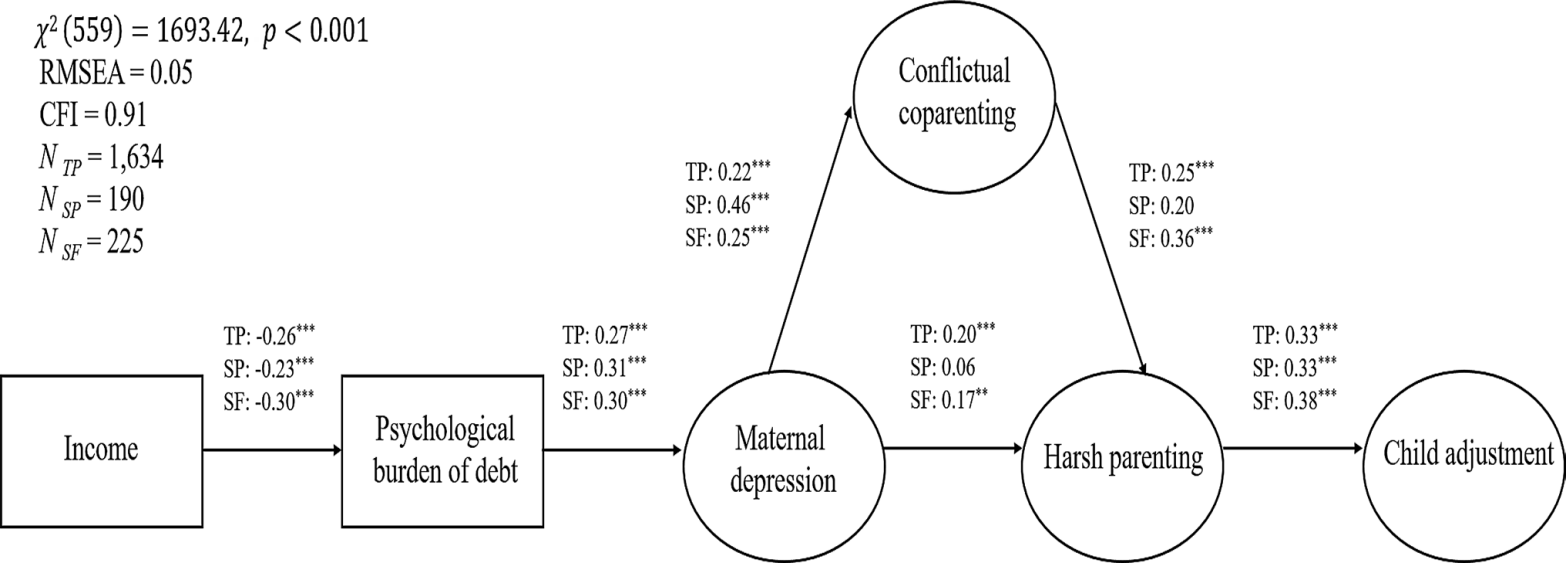
Multigroup structural equation model displaying standardized coefficients for the direct structural paths. Rectangles indicate manifest indicators; circles indicate latent constructs; error terms are omitted. *TP* Two-parent families, *SP* Single parents, *SF* Stepfamilies. ^***^p < 0.001 ^**^p < 0.01

Figure 4 summarizes model fit indices and standardized direct structural path coefficients for the MGA without equality constraints. The CFI was slightly lower compared to the SEM without subgroups, but the model fit was overall still acceptable. It can further be seen that the results for two-parent families and stepfamilies were largely in line with the findings outlined above. For single parents, there were no significant paths between maternal depressive symptoms and harsh parenting practices, as well as between conflictual coparenting (with the ex-partner) and harsh parenting practices. Yet mothers’ harsher parenting practices were nevertheless linked to higher levels of children’s adjustment problems for single parents as well. The association between maternal depressive symptoms and conflictual coparenting was particularly strong among single parents compared to two-parent and stepfamilies and the path between conflictual coparenting and harsh parenting practices seemed to be stronger for stepfamilies compared to two-parent families.

Lastly, all indirect paths were significant for two-parent families and stepfamilies (see Table 3), which indicates the presence of mediation specified in the conceptual model for these families. For single-parents, however, the following indirect structural paths were not significant: from income to mother’s harsh parenting practices and all indirect paths to child adjustment. This indicates that the mediation hypotheses specified in the conceptual model (i.e., between maternal depression, conflictual coparenting, and mother’s harsh parenting) were not fulfilled for single parents.

## Discussion

Our study contributes to the emerging literature on the distribution of debt across families—particularly by family structure—and potential ripple effects of the psychological burden related to debt on families. These issues are particularly timely in light of the increased availability to purchase various goods on installment plans, which families may be more likely to rely on in times of economic strain or uncertainty (Cooper & Pugh, 2020), and the global rise in the amount of household debt (Coletta et al., 2019). Germany, despite being among the richest industrialized economies worldwide (World Population Review, 2020), is no exception to this rule, which is reflected in its sizeable share of overindebted adults and families (STATISTA, 2020).

Because of well-documented social disparities in the accumulation of wealth by family structure (e.g., Bernardi & Mortelmans, 2018; Heintz-Martin & Langmeyer, 2019; Raley & Sweeney, 2020), we expected post-separation families to be more likely to have household debt compared to two-parent families. This hypothesis was partially fulfilled because we observed that only single parents, but not stepfamilies, were less likely to report having debt compared to two-parent families, which is in line with prior findings on the heightened poverty risk of single mothers (e.g., Chzhen & Bradshaw, 2012). This finding, and the lack of differences between stepfamilies and two-parent families, could be related to the privileged access to loans by banks and creditors for dual-earner households in contrast to single parents who would need to shoulder financial obligations to lenders by themselves. Relatedly, families with higher levels of income, who are likely to be dual-earner couples contributing to a joint accumulation of assets (Umberson & Thomeer, 2020), were also more likely to report having debt.

Our finding that respondents with both low and high levels of perceived economic deprivation were more likely to report having household debt compared to those with no perceived economic deprivation could further suggest that taking on debt served different purposes for these groups. It could be the case that for more affluent families, who are more likely to take on household debt in order to accumulate future assets and wealth (e.g., Aratani & Chau, 2010), such as by buying a house, making this investment buffers well-being (Dew, 2007; Dwyer et al., 2011). Lower-income families, however, may be pressed to take on debt or to use installment plans for smaller, more immediate purchases that do not necessarily yield any long-term returns (Bridges & Disney, 2004; Pfeiffer et al., 2016). Social disparities in the use and purpose for taking on household debt between these groups, which already became more accentuated during the Great Recession (Dunn & Mirzaie, 2016; Jenkins et al., 2013), could potentially grow further due to the recent COVID-19 pandemic. The pandemic increased rates of unemployment at least temporarily and is further likely to heighten families’ need for financial assistance (Settersten et al., 2020).

It is further likely that the distribution of these distinct types of debt varies by family structure (Hurst, 2011), which is in part related to processes of social selection and social causation (Umberson & Thomeer, 2020). For example, the significant interaction terms between family structure and perceived economic deprivation in our models indicated that, among both single parents and stepfamilies, those with high levels of perceived economic deprivation were more likely to report having debt. Among two-parent families, those with low levels of perceived economic deprivation were more likely to have debt. Our data did unfortunately not contain any information on the types of debt that families took on (cf. Dew, 2007). However, this pattern of results could suggest that two-parent families were those who took on debt for large-scale investments, whereas post-separation families were pressed to use debt for smaller, more immediate purchases. Unique characteristics of the German welfare state, such as its promotion of a more traditional two-parent norm, a shortage of childcare opportunities, and taxation that actively discourages both parents to work full-time (Grunow et al., 2018; Thévenon, 2011), may have also fostered differences in the distribution and use of debt by family structure in our sample. More specifically, lacking state support for mothers’ family-work-reconciliation may further elevate single mothers’ poverty risk in Germany because these mothers may be pushed into more precarious, and often lower-paying, marginal or part-time jobs. This may make them more prone to take on short-term debt, which can, in turn, diminish mother’s and children’s health, well-being, or educational and career aspirations (e.g., Duncan et al., 2012; Gaydosh & Harris, 2018; Shonkoff & Garner, 2012).

Because a large body of research has further focused on the adverse effects of financial strain on individuals’ well-being and family relations more broadly (e.g., Falconier & Epstein, 2010; Neppl et al., 2015; Park & Kim, 2018), our study aimed to specifically examine potential ripple effects of the psychological burden related to debt on families. In line with our conceptual model based on the FSM (Conger & Conger, 2002; Conger et al., 2010), results yielded that, for the subsample of families with accumulated household debt, there was a negative link between income and the perceived psychological burden of having debt. We further found a positive link between the perceived psychological burden related to debt and mothers’ depressive symptoms, which is in line with is in line with prior work on the association between financial strain due to debt and mental health issues (e.g., anxiety or depression; Bridges & Disney, 2010; Drentea & Reynolds, 2012; Jenkins et al., 2008). The positive links between maternal depressive symptoms and conflictual coparenting documented, for instance, by Williams (2018) and Tissot and colleagues (2017), as well as between maternal depression and harsh parenting practices (Choi & Becher, 2019), were also supported by our findings. Note, however, that due to the cross-sectional design of our underlying data, we were not able to disentangle the directionality of the associations between maternal depression, conflictual coparenting, and harsh parenting practices in contrast to prior studies using longitudinal data (e.g., Tissot et al., 2017). Conflictual coparenting was further positively associated with harsh parenting practices and harsh parenting practices were, in turn, negatively linked to child adjustment (Choi & Becher, 2019; McConnell et al., 2011; Waylen & Stewart-Brown, 2010). Taken together, these findings indicate that the FSM, which conceptualized the impact of financial strain more broadly, seems suitable to explain the ripple effects of the psychological burden related to debt on families as well.

We also expected the associations between the psychological burden of debt, parental dynamics, parent well-being, and child adjustment to be more aggravated for single parents and stepfamilies because of well-documented social disparities in health, well-being, and financial strain among post-separation parents (e.g., Burstrom et al., 2010; Heintz-Martin & Langmeyer, 2019; Pollmann-Schult, 2018). Even though results did not differ substantially between two-parent and stepfamilies, we did observe that, only for single parents, there were no significant direct or mediated paths between maternal depression and harsh parenting practices, as well as between conflictual coparenting and harsh parenting practices. This difference could be due to two reasons. First, single mothers may serve as gatekeepers in particularly conflictual coparenting relationships with a non-residential father by intentionally not letting these tensions spill over on their own parenting practices (Austin et al., 2013). Second, questions concerning conflictual coparenting were referring to the co-residential partner for two-parent families and stepfamilies, and only for single mothers to the non-residential father because single parents do not have a co-residential partner by definition. It could therefore be the case that these items measured slightly different dynamics because this relationship is likely to be more conflictual compared to that with a co-residential parent, which can affect maternal well-being considerably in addition to the adverse effect of household debt (Lamela et al., 2016). Alternatively, because the group of single mothers was the smallest group in our sample, it could be the case that our model was underpowered for this group to detect substantial effects for these complex associations.

## Limitations and Conclusion

This study has several limitations. First and as discussed above, our analyses were based on cross-sectional data. Consequently, the cross-sectional measures of debt, its potential psychological burden, and other family dynamics only provided a single snapshot at the time of data collection that do not allow to infer any causal claims. For example, it is possible that higher levels of maternal depression also lead to higher ratings on the perceived burden of debt rather than the other way around, as our model suggested. We further acknowledge the possibility of self-selection into debt by parents’ mental health (Umberson & Thomeer, 2020). However, our analyses were theoretically grounded in the well-established FSM and future studies will need to disentangle the directionality of debt-specific effects based on suitable longitudinal data.

Second, due to secondary data limitation issues, we had to rely solely on mother’s report on all of our measures. Because of more traditional gender norms and role distributions between parents fostered by policies of the German male breadwinner model (Grunow et al., 2018; Thévenon, 2011), fathers are more likely to earn more money compared to mothers and are more likely to pay child support in post-separation families. It would therefore have been beneficial to also have fathers’ perspective on household debt and its potential psychological burden (cf. Ponnet et al., 2016). As previously stated, information on the types of debt (e.g., for future investments vs. daily consumption) were also not available, which are likely affect how families cope with having debt (Dwyer et al., 2011). We therefore included multiple indicators of families’ socio-economic situation, such as income and perceived economic deprivation, at different points of the analyses to adjust for social disparities in the likelihood to have certain types of debt (e.g., low-income households are less likely to take on debt for future investments). On other measures concerning the quality of family interactions, such as the parental coparenting relationship or mothers’ harsh parenting practices, having to rely on mothers’ report only rather than including fathers’ perspective, or even that of a more neutral observer, could have also introduced some degree of social desirability bias on the ratings.

Third, the results of our study may not be generalizable to other contexts without caution for two reasons. On the one hand, the German welfare state has a social security net with high levels of financial support for all families regardless of financial need above the OECD average (Thévenon, 2011). Thus, the perceived psychological burden of having debt and its ripple effects on parental well-being, parenting dynamics, and child adjustment may be even more aggravated in other welfare contexts with less state support in the case of financial need (e.g., in the more market-oriented Anglo-Saxon countries). On the other hand, certain types of debt, such as credit card debt, are not as relevant and widespread in Germany because of the more strongly regulated access to credit cards and hire purchases. Yet particularly this kind of unsecured debt is likely to be taken on for means of daily consumption rather than future investments, which are, in turn, likely to exacerbate the psychological toll related to debt and to dampen well-being.

Despite these limitations, our study contributes to the literature on the distribution of household debt across family structure by highlighting that both post-separation and two-parent families were affected by debt, but likely for different reasons. Differential usages of debt in terms of future investments vs. short-term consumption may widen the economic divide between families and by family structure, which could further magnify due to unprecedented rates of unemployment and underemployment during the recent COVID-19 pandemic. We conclude that implementing programs that improve financial literacy skills, as well as improvements in mothers’ family-work-reconciliation, are particularly important for women after a separation or divorce. Being able to make informed decisions on financial issues and to avoid the accumulation of more risky short-term debt, if possible, may protect these mothers from financial hardship and its adverse psychological toll. Relatedly, our study further provided insights into potential ripple effects of the psychological burden related to debt on maternal well-being, parenting dynamics, and child adjustment and by family structure. Families across the social strata were adversely affected by the psychological toll related to having household debt and these associations were largely in with the FSM, which conceptualized the impact of financial strain on families more broadly. One exception was the group of single mothers, who may have served as gatekeepers by dampening the effect of potentially debt-induced detriments to their own mental well-being and conflictual coparenting relationships with a non-residential father on mothers’ harsh parenting practices (Fagan & Kaufman, 2015). This could indicate that investing in programs targeting the often highly conflictual coparenting relationship among post-separation parents (Eira Nunes et al., 2020), particularly among single parents, could relieve some degree of parental role strain from these mothers and, in turn, increase the use of more supportive parenting practices (Hakvoort et al., 2012).
